# Optimization of miR-22 expression cassette for rAAV delivery on diabetes

**DOI:** 10.1186/s43556-021-00063-y

**Published:** 2022-01-05

**Authors:** Li Yang, Wenya Du, Zhaoyue Zheng, Li Wang, Lin Xiao, Qingzhe Yang, Qiukui Hao, Jiao Zhou, Jintao Du, Jun Li, C. Alexander Valencia, Birong Dong, Hoi Yee Chow, Xianghui Fu, Biao Dong

**Affiliations:** 1 Department of Geriatrics and National Clinical Research Center for Geriatrics, West China Hospital, Sichuan University, Chengdu, Sichuan Province China; 2 State Key Laboratory of Biotherapy, West China Hospital, Sichuan University, Chengdu, Sichuan Province China; 3 Department of Otorhinolaryngology Head & Neck Surgery, West China Hospital, Sichuan University, Chengdu, Sichuan Province China

**Keywords:** Recombinant adeno-associated virus, MicroRNA, Expression cassettes, MiR-22, Insulin

## Abstract

MicroRNA-22 (miR-22) was suggested to be important for type 2 diabetes but its functions for this disease remained unclear. Recombinant adeno-associated virus (rAAV)-mediated miR delivery is a powerful approach to study miR functions in vivo, however, the overexpression of miR-22 by rAAV remains challenging because it is one of the most abundant miRs in the liver. In this study, a series of expression cassettes were designed and compared. It was shown that different lengths of primary miR-22 were overexpressed in HEK293 and HeLa cells but the longer ones were more efficiently expressed. miR-22 may be placed in either introns or the 3′ UTR of a transgene for efficient overexpression. RNA polymerase III or II promoters were successfully utilized for miR expression but the latter showed higher expression levels in cell lines. Specifically, miR-22 was expressed efficiently together with an EGFP gene. After screening, a liver-specific TTR promoter was chosen to overexpress miR-22 in diabetic mice fed a high-fat diet. It was shown that miR-22 was overexpressed 2-3 folds which improved the insulin sensitivity significantly. The approach utilized in this study to optimize miR overexpression is a powerful tool for the creation of efficient rAAV vectors for the other miRs.

**Supplementary Information:**

The online version contains supplementary material available at 10.1186/s43556-021-00063-y.

## Introduction

MicroRNAs (miRs) are single-stranded non-coding RNAs, 20- to 24-nt long, which regulate gene expression post-transcriptionally by binding to 3′ untranslated region (3′ UTR) of their target mRNAs [1]. After base-pairing binding, the targeted mRNAs are degraded or destabilized, resulting in the reduction of targeted proteins. In the cell nucleus, single-strand primary microRNAs (pri-miRs) are generally transcribed by RNA polymerase II. Then, pri-miRs form a hairpin loop structure, which is cleaved by Drosha, resulting in short hairpin precursor microRNAs (pre-miRs). Pre-miRs are released into the cytoplasm and further cleaved into double-stranded ~ 22-nt long mature miRs by Dicer. After strand separation, a matured single-stranded miR is incorporated into the silencing complex miRISC.

To study the biological functions of miRs, pri-miRs or pre-miRs, they are usually expressed under the control of desired promoters. RNA polymerase III promoters, namely H1 and U6, and RNA polymerase II promoters, such as the constitutive CMV promoter, are both used for this purpose [2], while the former is chosen more often for delivering various kinds of short RNAs. In the expression cassettes, pri-miRs have been put in the intron or 3′ UTR of a transgene [3–6], while introns were usually used to stabilize mRNA and enhance the transgene overexpression [7, 8]. Although miRs are found to play important roles in many diseases, only a few studies have reported the systematic optimization of the expression cassettes to understand their functions.

miR-22 is a 22-nt long RNA and plays important roles in many physiological and pathological processes, including glucose metabolism [9, 10], vascular smooth muscle cell differentiation [11], and cancers progression [12]. Interestingly, recent studies indicates that miR-22 can regulate gluconeogenesis, which mainly occurs in the liver and is considered as a hallmark of type 2 diabetes [9, 13–15]. Of note, the role of miR-22 in diabetes is still ambiguous. It was shown that an miR-22 inhibitor improved glucose tolerance and insulin sensitivity in db/db mice, while miR-22 mimics aggravated glucose intolerance in C57BL/6 J mice [16]. The miR-22 inhibitor, APT-110, decreased the bodyweight and improved glucose tolerance in high-fat diet (HFD) mice [17]. In contrast, a recent study showed that miR-22 mimics improved insulin sensitivity in mice with gestational diabetes mellitus [18]. Additionally, it was reported that miR-22 deficiency did not affect the glucose tolerance and insulin resistance in mice fed a HFD [19]. However, another study showed that miR-22 deletion in mice increased the bodyweight and fat accumulation, and deteriorated glucose intolerance and insulin resistance under HFD condition [20]. It was also noted that miR-22 is one of the most abundant miRs in the liver [20], which could make it difficult to be overexpressed.

rAAV vectors are chosen as delivery vehicles to study miR functions in vivo because these viral vectors are nonpathogenic, safe, and highly efficient at transducing many types of cells [21]. When delivered by rAAV vectors, most of miRs were overexpressed under the control of type II promoters such as EF1α or CMV [22, 23]. And pri-miRs could be put in the intron or 3′ UTR of a transgene for miRs overexpression in rAAV vectors [3, 4, 6]. It may be necessary to optimize expression cassette when a transgene and a miR need to be co-expressed utilizing one vector because the packaging capacity of rAAV is limited. Hundreds of AAV serotypes have been found and they show different tissue tropisms. According to the difference in ITR structures, which are the packaging signals and are located at both ends of the expression cassettes, rAAVs can be classified as single-stranded (ss) vectors and self-complementary (sc) vectors [24]. The packaging limits for these two vectors are around 5.0 kb and 2.5 kb [25, 26], respectively. Compared with ssAAV vectors, scAAV vectors are more efficient at transgene overexpression. AAV8 is the most commonly used serotype to study gene functions in the mouse liver [27]. Kota J et al demonstrated that scAAV8-mediated miR-26a overexpression can effectively inhibit cancer cell proliferation [3].

Since the function of miR-22 on type 2 diabetes was ambiguous, it needs to be overexpressed specifically in the mouse liver to investigate the resulting effects on glucose metabolism, the hallmark for identifying diabetes. To this end, the promoter and miR-22 sequence needs to be optimized before the overexpression cassette is delivered by an rAAV vector in serotype 8 which has a strong liver tropism after intravenous injection.

## Results

### Effect of varying lengths on pri-miR-22 overexpression in cells

**Fig. 1:**
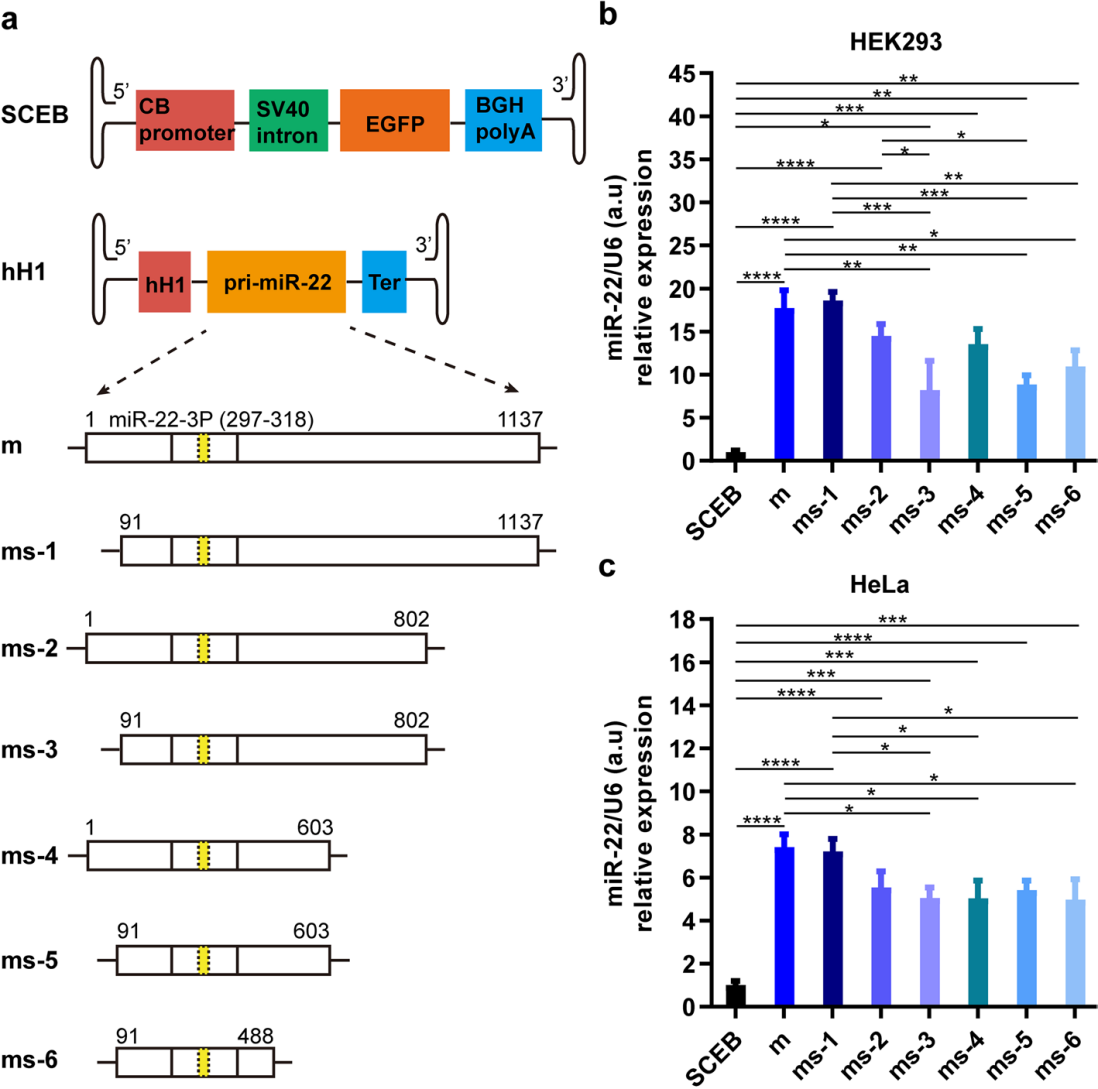
Expression comparisons by varying pri-miR-22 lengths in cells. **a** Schematic diagram of the rAAV vector plasmids containing different lengths of pri-miR-22 driven by the hH1 promoter. CB, chicken β-actin promoter with the CMV enhancer; hH1, human H1 promoter; Ter, TTTTTT sequence, and the termination signal for miRs. miR-22-3p was indicated in yellow and ms1-6 represented different lengths of shortened pri-miR-22. **b** Expression levels of different lengths of miR-22 in HEK293 cells. **c** Expression levels of different lengths of miR-22 in HeLa cells. All data were represented as Mean ± SD, and error bars were based on triplicated samples. *P < 0.05, **P < 0.01, ***P < 0.001 and ****P < 0.0001

miR-22 is relatively abundant in cells and its overexpression may be difficult compared with those with lower expression levels such as miR-199a [20, 28–30]. Since the length of pri-miRNA may affect miRNA expression and rAAV has a limited packaging capacity [25, 26, 31], it was necessary to identify the appropriate length of the pri-miR-22. To this end, a series of rAAV plasmid vectors containing different lengths of pri-miR-22 were designed, all of which were under the control of human H1 (hH1) promoter (Fig. 1a). The SCEB vector was a single-stranded rAAV vector plasmid expressing only EGFP, which was used in this study as a negative control. Its expression cassette was composed of a CB promoter, an SV40 intron, an EGFP ORF, and a BGH polyA. The whole expression cassette was flanked by two ITRs which are the signals for rAAV packaging.

The plasmid vectors were transfected into HEK293 cells (Fig. 1b) and HeLa cells (Fig. 1c), and the expression levels of miR-22-3p, the major form of mature miR-22, were measured using RT-qPCR. It was shown that all miR-22 constructs were significantly overexpressed in both cells compared with the negative control. It was demonstrated that the length of pri-miR-22 did affect its expression, and that the two longer forms of pri-miR-22, m (1137 bp) and ms-1 (1047 bp), had relatively higher expression levels than other shorter forms. The shortest form ms-6 (398 bp) may be used for overexpression, but the expression level was significantly lower than the longest one in both cell lines. The 1137 bp form (m) of pri-miR-22 was chosen for further study. It was noted that ms-2 had a significantly higher expression level than ms-3 and ms-5 in HEK293 cells while there were no significant differences among their expression levels in HeLa cells. This suggest that the overexpression of miR-22 may be cell line dependent.

### Effect of Pol II promoter and Pol III promoters on miR-22 overexpression in cells

**Fig. 2:**
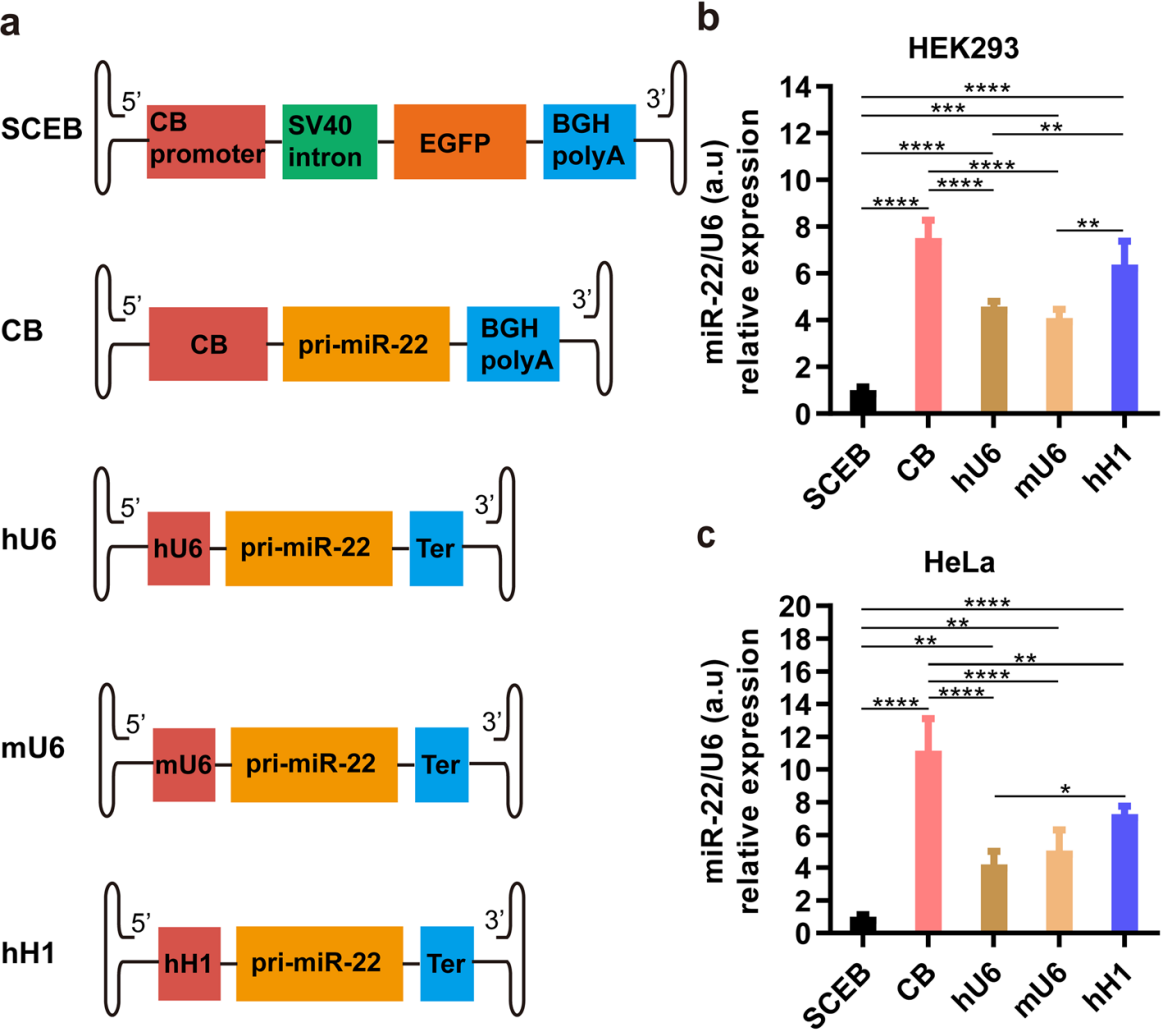
miR-22 expression comparisons driven by the Pol II promoter (CB) and Pol III promoters (hU6, mU6, and hH1). **a** Schematic pictures of miR-22 expression cassettes in the rAAV vector plasmids with different promoters. CB, chicken β-actin promoter with CMV enhancer; hU6, human U6 promoter; mU6, mouse U6 promoter; hH1, human H1 promoter; Ter, TTTTTT sequence, the termination signal for miRs. **b** miR-22 expression levels driven by different promoters in HEK293 cells. **c** miR-22 expression levels driven by different promoters in HeLa cells. *n* = 4, error bars represent the standard deviation of the mean. *P < 0.05, **P < 0.01, ***P < 0.001, and ****P < 0.0001

To compare the expression of miR-22 under different types of promoters, three Pol III promoters, including human U6 (hU6), mouse U6 (mU6), and human H1 promoter (hH1), and one Pol II promoter (CB promoter) were tested (Fig. 2a). It was shown that all promoters may be used for miR-22 overexpression, and that the expression levels driven by these promoters were at least 4-fold higher relative to the control vector SCEB in HEK293 cells (Fig. 2b) and HeLa cells (Fig. 2c). miR-22 expression levels driven by the CB promoter were the highest in both cells, and the second highest was the hH1 promoter.

### Effect of intron position on miR-22 overexpression in cells

**Fig. 3:**
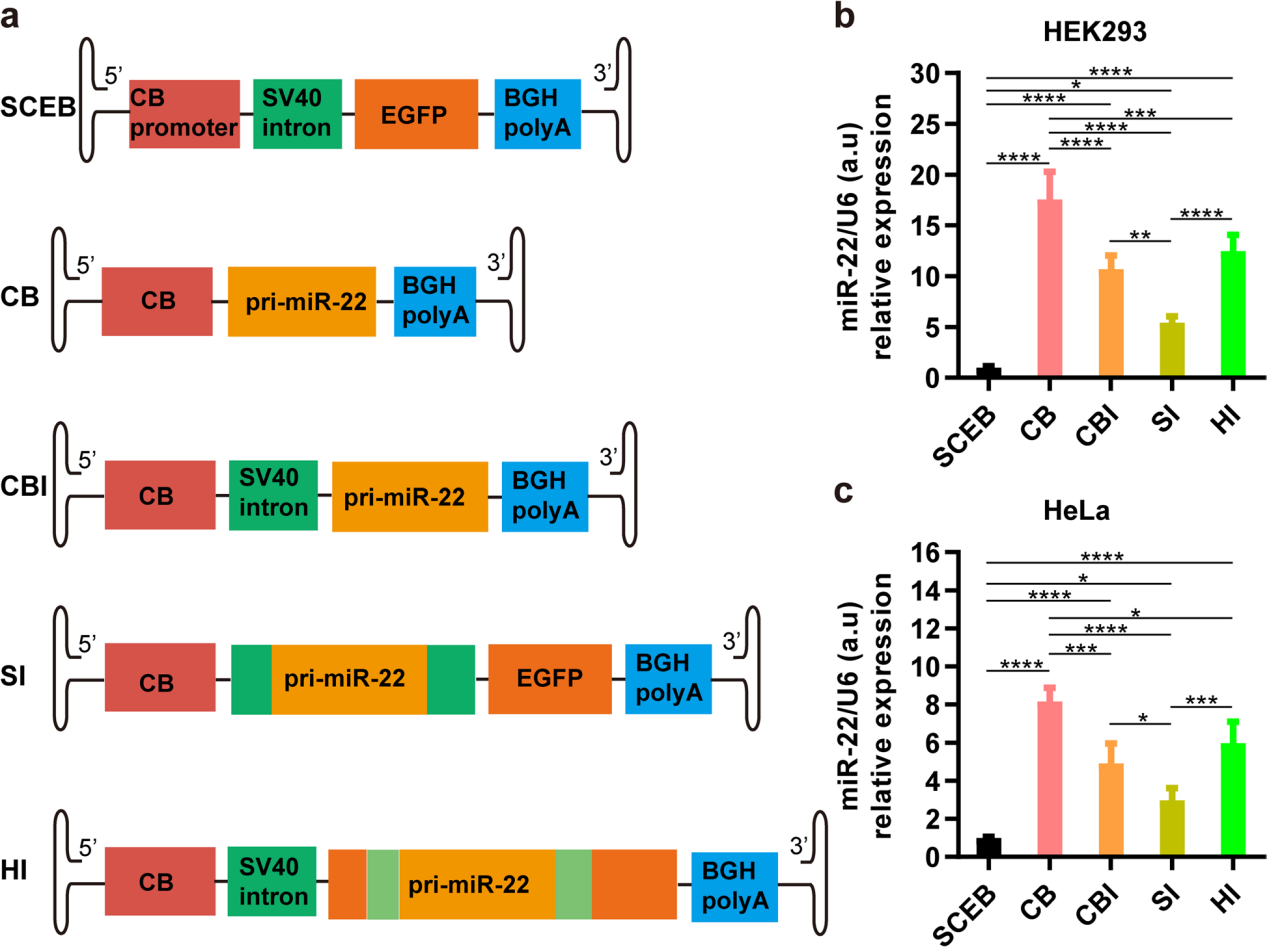
Effect of the intron position on miR-22 overexpression in cells. **a** Schematic pictures of miR-22 expression cassettes in the rAAV vector plasmids with pri-miR-22 at different positions relative to an intron or introns. Two boxes in light green on both sides of pri-miR-22 in HI vector indicated separate parts of another intron named hCG intron from the *hCG* gene. **b** miR-22 expression levels in HEK293 cells. **c** miR-22 expression levels in HeLa cells. n = 4, error bars represent the standard deviation of the mean. *P < 0.05, **P < 0.01, ***P < 0.001, and ****P < 0.0001

To study the effect of intron cassette location on miR overexpression, the SV40 intron was utilized to design a series of vectors in which miR-22 was directly driven by CB promoter (CB), behind the intron (CBI), inside the intron (SI), or inside the EGFP gene flanked by another hCG intron (HI) (Fig. 3a). The transfection results showed that miR-22 was overexpressed by all constructs in both HEK293 cells (Fig. 3b) and HeLa cells (Fig. 3c). miR-22 without any intron behind the promoter had the highest expression levels, indicating that the effect of the SV40 intron on miR-22 expression was opposite to that on transgene expression [8]. The miR-22 expression level was higher when it was located behind the intron (CBI) than inside the intron (SI). Interestingly, miR-22 was overexpressed at relatively higher levels when it was flanked by a separated hCG intron inside the EGFP expression cassette. However, there were no significant differences between miR-22 expression levels of the CBI group and HI group in both HEK293 cells and HeLa cells.

### Effect of EGFP expression cassette on miR-22 overexpression in cells

When a transgene and a miR need to be co-expressed, it is essential to discern the potential expression effects that the transgene and miR may have using one expression vector. It was shown that miR-22 was co-expressed with the EGFP gene (Fig. 3). Thus, the utilization of the EGFP expression cassette was continued to further investigate how the transgene expression cassette affected miR-22 expression. Another advantage of using EGFP is that the infected cells could be tracked by cell sorting.

**Fig. 4:**
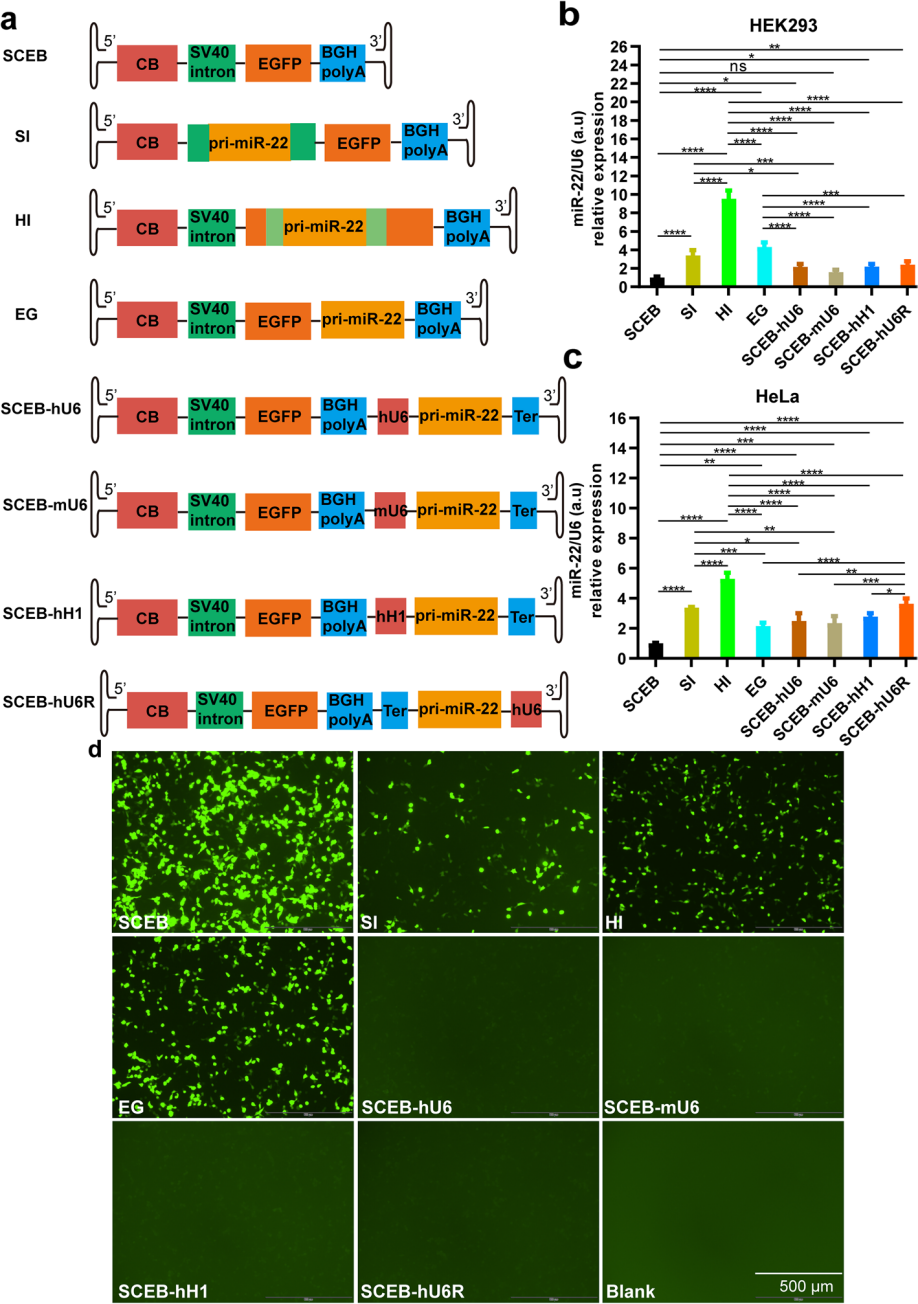
miR-22 expression comparisons dependent on or independent of EGFP expression cassettes. **a** Schematic pictures of miR-22 and EGFP expression cassettes in rAAV vector plasmids. CB, chicken β-actin promoter with CMV enhancer; hH1, human H1 promoter; Ter, TTTTTT sequence, the termination signal for miRs. Two boxes in light green on both sides of pri-miR-22 in HI vector indicated separate parts of another intron named hCG intron from *hCG* gene. **b** miR-22 expression levels in HEK293 cells. **c** miR-22 expression levels in HeLa cells. **d** EGFP expression in HEK293 cells. n = 4, error bars represent the standard deviation of the mean. *P < 0.05, **P < 0.01, ***P < 0.001, and ****P < 0.0001

To test EGFP and miR-22 co-expression, a series of rAAV vectors were designed including miR-22 expression cassettes dependent on EGFP expression cassettes (SI, HI, and EG, Fig. 4a) or independent of EGFP expression cassettes for comparisons (SCEB-hU6, SCEB-mU6, SCEB-hH1 and SCEB-hU6R, Fig. 4a). In the EG vector, miR-22 was placed at the 3′ UTR of the EGFP transgene which was a popular reported position [6]. SCEB-hU6 and SCEB-hU6R were two vectors that had the reverse miR-22 overexpression cassette. The transfection results showed that all constructs overexpressed miR-22. HI had the highest expression levels among SI, HI, and EG vectors for miR-22 overexpression in both HEK293 and HeLa cells (Fig. 4b and c). However, the EGFP expression level in HEK293 cells using the EG vector was higher than SI and HI. Compared with the control vector without miR expression (SCEB), the EGFP expression levels in all other vectors decreased, suggesting that miR-22 overexpression disrupted EGFP expression (Fig. 4d). EGFP expression in the EG vector was higher than other vectors except for SCEB, but the miR-22 expression level by this vector was significantly lower than that seen with the HI vector in both HEK293 cells (*P* < 0.0001) and HeLa cells (*P* < 0.0001). When the two expression cassettes were independent of each other in one vector, EGFP expression was largely decreased and miR-22 expression levels also declined compared with the HI vector (Fig. 4b and c). There was no significant difference between the expression levels of miR-22 using SCEB-hU6 and SCEB-hU6R plasmids in HEK293 cells, while SCEB-hU6R showed higher expression than SCEB-hU6 in HeLa cells.

### rAAV-mediated overexpression of miR-22 in mouse livers

**Fig. 5:**
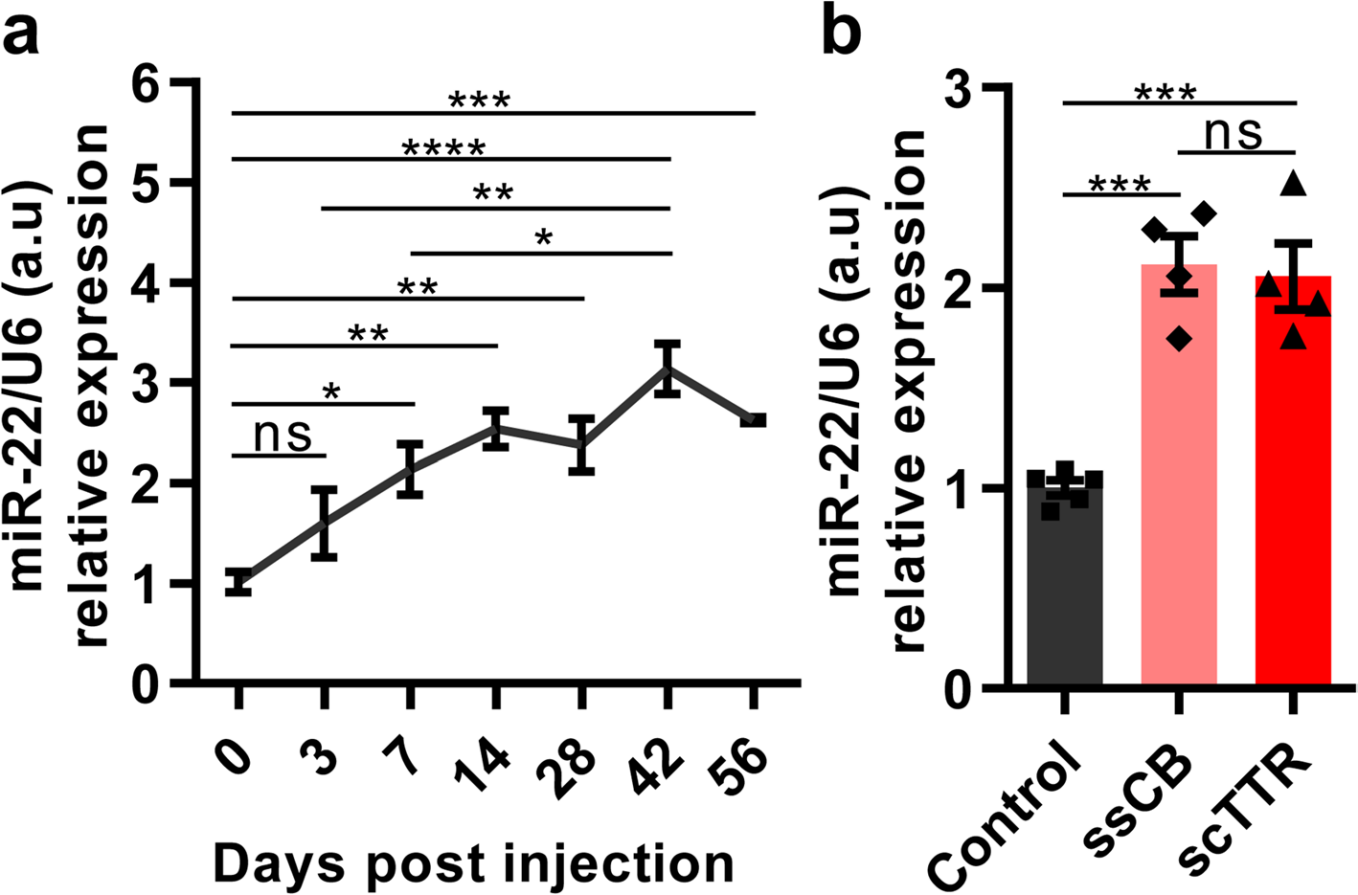
rAAV-mediated overexpression of miR-22 in mouse livers. **a** A miR-22 overexpression time course in the mouse livers mediated by the single-stranded rAAV vector, AAV8-CB-miR-22 vector (ssCB). **b** Comparison of miR-22 expression levels in the livers of ssCB and scTTR mice. n = 4 ~ 5, all data were reported as mean ± SEM. *P < 0.05, **P < 0.01, ***P < 0.001, ****P < 0.0001 and ns represented not significant

To study the biological functions of miR-22 in mouse livers, we characterized its expression levels at different time points post-injection of a single-stranded rAAV vector ssCB (Fig. 5a). The vector was packaged in the rAAV8 serotype which has a high infection tropism to the mouse liver [27]. To minimize the possible errors of the analysis due to mouse manipulations, a negative control vector, SCEB, was used.

It was shown that miR-22 was significantly overexpressed after 7 days post-injection (*P* = 0.0270, Fig. 5a). miR-22 expression levels were dramatically up-regulated on day 14 (*P* = 0.0012), 28 (*P* = 0.0043), 42 (*P* < 0.0001), and 56 (*P* = 0.0006) post-injection compared with SCEB group mice, while there were no significant differences among the miR-22 expression levels at these four time points. This result suggested that miR-22 expression nearly reached its expression peak 14 days post-injection and with expression lasting for a long time in vivo.

The liver is one of the major organs where miR-22 plays important functions, thus, we tested its overexpression in a tissue-specific manner. Based on the result that miR-22 expression is driven by Pol II promoter (Fig. 2), a liver-specific TTR promoter was chosen and a self-complementary rAAV vector (scTTR) was constructed. After tail vein injections of the scTTR vector, mouse livers were collected to check the miR-22 expression 14 days post-injection by RT-qPCR. It was shown that miR-22 was significantly overexpressed using the scTTR vector compared with the control (*P* < 0.001, Fig. 5b).

### Biological functions of miR-22 overexpression on bodyweight and insulin sensitivity in HFD mice

**Fig. 6:**
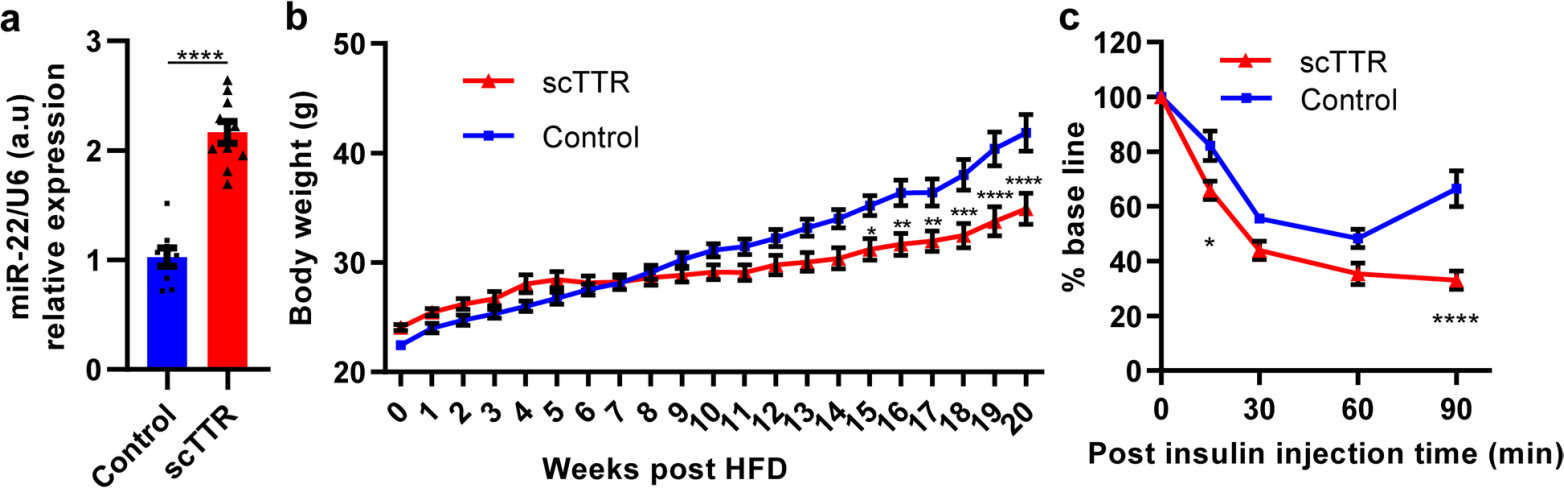
Effect of hepatic miR-22 overexpression on bodyweight and insulin sensitivity in HFD mice. **a** miR-22 expression levels in the mouse livers 37 weeks post scTTR or SCEB injection and HFD treatment for 35 weeks (*n* = 9 ~ 10). **b** Bodyweight of the two groups of mice treated with scTTR or SCEB vectors (*n* = 10 for each group). **c** The insulin tolerance test (ITT) of two groups of mice treated with scTTR or SCEB vectors (*n* = 8 for each group). All data were reported as mean ± SEM. *P < 0.05, **P < 0.01, ***P < 0.001, ****P < 0.0001

The function of miR-22 on insulin sensitivity were controversial [16, 18–20]. To address this question, we investigated whether miR-22 overexpression affected bodyweight and insulin sensitivity in obese and diabetic mice induced by a high-fat diet (HFD). It was found that miR-22 was significantly overexpressed in mice with HFD treatment started 2 weeks after scTTR injection and lasting for 35 weeks compared with the SCEB group (*P* < 0.0001, Fig. 6a). The bodyweight of HFD mice treated with scTTR rAAV vector significantly decreased after 15 weeks, and the mean bodyweight decreased 4, 4.7, 4.45, 5.55, 6.64, and 6.92 g at 15, 16, 17, 18, 19, and 20 weeks, respectively (Fig. 6b). Importantly, the insulin tolerance test showed that miR-22 overexpression increased insulin sensitivity at 15 min (*P* = 0.0124) and 90 min (*P* < 0.0001) after insulin administration in HFD mice (Fig. 6c). Altogether, liver-specific overexpression of miR-22 mediated by rAAV vector decreased the bodyweight and enhanced insulin sensitivity in HFD mice.

## Discussion

In this study, to investigate the possible function of miR-22 on insulin sensitivity in the liver of diabetic mice, the expression cassettes suitable for rAAV delivery were screened and a liver-specific TTR promoter was selected.

In previous studies to examine the expression of miRs, both RNA polymerase II and polymerase III promoters have been utilized and the pri-miRs were placed in the intron or 3′ UTR of the transgene expression cassette [2–6]. In recent reports, few systematic comparisons using different expression cassettes for the same miR were described. In this study, it was shown that both kinds of promoters overexpressed miR-22, and that the RNA polymerase II promoter, CB, was stronger than RNA polymerase III promoters in both HEK293 and HeLa cell lines (Fig. 2b and c).

Instead of the regularly used constitutive RNA polymerase II promoter, such as CB or CMV, the tissue-specific TTR promoter was used in this in vivo study. The TTR promoter has been widely used for the overexpression of hepatic genes such as coagulation of factor IX [32–34], but rarely used for the overexpression of miRs [35, 36]. We hypothesized that the functions of miR-22 in the liver may be studied more accurately by using a TTR promoter, and this hypothesis was supported by the expression data (Fig. 6). rAAV gene therapy using miRs may be the next generation of therapies for some acquired diseases that are difficult to treat such as Huntington’s disease and heart failure. Our results suggested that tissue-specific promoters may be utilized to improve the safety of such therapy.

miR expression cassettes should be optimized depending on the selected miRs. miR-22 is one of the most abundant miRs in the liver cells, so its overexpression is relatively difficult compared with rare ones. It was shown that miR-22 may be overexpressed up to 2-16 folds in vitro (Figs. 1, 2, 3, and 4) and 2-4 folds in vivo (Figs. 5 and 6). However, the rare miR-199a was overexpressed more than 1000-fold increase in vitro using the same expression cassette (Supplementary Fig. 1). Also, the expression cassettes, such as SCEB-hU6 that was not efficient for miR-22 overexpression, were efficient for miR-199a overexpression (Fig. 4 and Supplementary Fig. 1).

We utilized the EGFP reporter gene to demonstrate that miRNA may be co-expressed with another gene (Fig. 4). We may take advantage of these vectors to deliver a therapeutic gene and a miRNA concurrently in human gene therapy strategies. For preclinical studies, EGFP signals may be used to trace cells having miRNA expression by fluorescent microscope or collected by sorting.

miR-22 is important for type 2 diabetes, but the relationship between miR-22 and type 2 diabetes remained to be elucidated. Kaur et al predicted that miR-22 had 705 gene targets from two databases of TargetScan and miRanda [14]. Besides, type 2 diabetes was reported to be associated with multiple miRNAs [37–39]. The previous studies reported the opposite conclusions on miR-22 function on glucose metabolism [16–20]. In this study, diabetic mouse model was induced by HFD (Supplementary Fig. 2). It was shown that overexpression of miR-22 in the liver decreased the bodyweight and increased insulin sensitivity in HFD-induced obese and diabetic mice (Fig. 6). This result indicated that rAAV gene therapy using pri-miR-22 may be useful as a type 2 diabetes treatment. However, although a similar increase in miR-22 was induced by rAAV vectors in the livers of both CD (conventional diet) and HFD mice (Fig. 5b and Fig. 6a), it is of importance to clarify whether fatty liver may affect the efficiency of rAAV-mediated gene expression in the future.

Altogether, the expression cassettes for miR-22 were systematically investigated, and the design and results help to clarify the function of miR-22 in diabetic mice.

## Material and methods

### Construction of rAAV vector plasmids

rAAV vector plasmids with different miR-22 expression cassettes were constructed for expression comparisons. The longest pri-miR-22 sequence used in this study contained *Mus musculus* miR-22 partial gene exon regions (NR_030711.1, exon 1: 1 ~ 217; exon 2: 218 ~ 382; exon 3: 383 ~ 1766), and was obtained by reverse transcription polymerase chain reaction (RT-PCR) using mouse liver RNA as the template. The detailed creation process of related plasmids and the primers were shown in the supplemental information.

### Package and purification of rAAV vectors

Three rAAV vectors, ssAAV8-CB-miR-22-BGH polyA (ssCB), scAAV8-TTR-miR-22-BGH polyA (scTTR), and a negative control vector ssAAV8-CB-SV40 intron-EGFP-BGH polyA (SCEB) were packaged using the triple plasmids transfection method in HEK293 cells as described previously and were purified by two rounds cesium chloride ultracentrifugation [40]. The titers of rAAV vectors were determined by qPCR using the miR-22 forward primer (5′ -AGCCTACATTCAAGGTAATC- 3′) and reverse primer (5′ -GCTTGTTGTATTATGATCAG- 3′).

### Cell culture and transfection

HEK293 cells and HeLa cells were obtained from ATCC and cultured in DMEM media containing 10% FBS and 1% Penicillin-Streptomycin. Cells were incubated at 37 °C with 5% CO_2_.

Transfection was performed in a 12-well plate format. Briefly, the cells were grown overnight until the confluency was around 70%, then the mixture of 0.5 μg plasmid and 1.5 μL of PolyJet (SignaGen Laboratories, Maryland) was added to each well according to the manufacturer’s protocol. The cells were transfected for each plasmid in triplicate and the miR-22 expression comparisons were repeated three times. The transfected cells were collected 24 h post-transfection.

### RT-qPCR

TRIzol (Invitrogen, Carlsbad) was utilized for total RNA isolation. cDNAs were obtained using 2 μg high-quality RNAs as templates in a 10 μL system according to the manufacturer’s protocol (Invitrogen, Carlsbad). The reactions were then placed on a PCR instrument at 37 °C for 50 min, followed by incubation at 70 °C for 15 min to obtain cDNAs.

Each cDNA sample was diluted 40-fold in nuclease-free water for a qPCR reaction to detect miR-22 and U6 snRNA separately in duplicate. A reaction in a 10 μL total volume containing 5 μL of SYBR mix (TransGen Biotech, Beijing), 4.7 μL of diluted cDNA sample, and 0.3 μL of 10 μM miR-22-3P primer set (GenePharma, Shanghai) or U6 snRNA primer set (GenePharma, Shanghai) was added into PCR tubes (Bio-Rad, Hercules) and the qPCR reaction was performed on an Applied Biosystems instrument. The qPCR procedure was 95 °C for 2 min, followed by 40 cycles of 95 °C for 5 s and 60 °C for 10 s. U6 snRNA expression was selected as an internal reference, and the 2^^-ΔΔCT^ method was used to quantify the relative expression levels of miR-22 [11].

### Animals

Approximately 6-8 weeks old C57BL/6 male mice (Vital River Laboratory Animal Technology, Beijing) were maintained on a 12-h light-dark cycle.

Mice were treated with different rAAV vectors via tail vein injections. The injection dosage of different rAAVs was 4 × 10^11^ genome copies (GC) for each mouse. The liver tissues were collected for expression profiling by RT-qPCR, and the negative control group mice were injected with the SCEB vector. Livers of mice injected with scTTR rAAV vector were also collected after 2 weeks post-injection and sacrificed for miR-22 expression measurements. The time course to profile miR-22 expression in mice treated with ssCB vector was explored on 3 days, 1 week, 2 weeks, 4 weeks, 6 weeks, and 8 weeks post-injection by sacrificing 5 mice at each time point.

Mice treated with the scTTR rAAV vector and the negative control vector were fed a high-fat diet (Research Diets, New Brunswick) 2 weeks after rAAV vectors injection. Livers of mice with rAAV vectors fed a high-fat diet for 35 weeks were collected for measuring miR-22 expression levels.

### Insulin tolerance test (ITT)

Mice treated with the scTTR rAAV vector and the negative control vector were fed a high-fat diet for 31 weeks. ITT was performed after the mice fasted for 6 h. The bodyweight and fasting glucose levels were measured before an insulin injection. Then, mice were administered insulin via an intraperitoneal injection, and the dosage of insulin was 1 U/kg bodyweight. Blood glucose levels were measured at 15, 30, 60, and 90 min after the insulin injection.

### Data analysis

GraphPad Prism 8.0.1 was used for data statistical analysis. Adobe Illustrator CS5 was used for figure production. All in vitro data were compared with every other group and were shown as mean ± SD, while all in vivo data were shown as mean ± SEM. Significant differences among the miR-22 expression levels of different groups in in vitro experiments were calculated by Ordinary One-Way ANOVA analysis followed by Turkey’s post hoc test. Significant differences among the miR-22 expression levels at different time points after rAAV vectors injection, and significant difference among the miR-22 expression levels of three groups in in vivo study were both calculated by Ordinary One-Way ANOVA analysis followed by Turkey’s post hoc test. The significant difference between the miR-22 expression levels of scTTR group and the negative control group was calculated by a two-tailed Student’s t-test. Significant differences between the scTTR group and the control group on the bodyweight and blood glucose levels at different time points were calculated by a Two-Way ANOVA analysis followed by Sidak’s post hoc test.

## Supplementary Information

**Additional file 1: Supplementary Fig. 1. **Comparison of the expression levels of miR-199a in different expression cassettes. Supplementary Fig. 2 Blood glucose and GLUT2 mRNA expression levels in the liver of mice.** Supplementary Table 1. **The primers for the construction of miR-22 expression cassettes.

## Data Availability

The data are available from the corresponding authors upon reasonable request.

## Abbreviations

rAAV: Recombinant adeno-associated virus
ssAAV: Single-stranded AAV
scAAV: Self-complementary AAV
UTR: Untranslated region
TTR: Transthyretin
miR: MicroRNA
miRISC: miRNA-induced silencing complex
HFD: High-fat diet
CD: Conventional diet
ITT: Insulin tolerance test
