# Supplementary material for: Optimization of miR-22 expression cassette for rAAV delivery on diabetes
Source: Mol Biomed. 2022 Jan 5;3:1. doi: 10.1186/s43556-021-00063-y (PMC8727650; doi:10.1186/s43556-021-00063-y)
Supplement: Supplementary file 1 — Additional file 1: Supplementary Fig. 1. Comparison of the expression levels of miR-199a in different expression cassettes. Supplementary Fig. 2 Blood glucose and GLUT2 mRNA expression levels in the liver of mice. Supplementary Table 1. The primers for the construction of miR-22 expression cassettes. [file 43556_2021_63_MOESM1_ESM.docx]

**
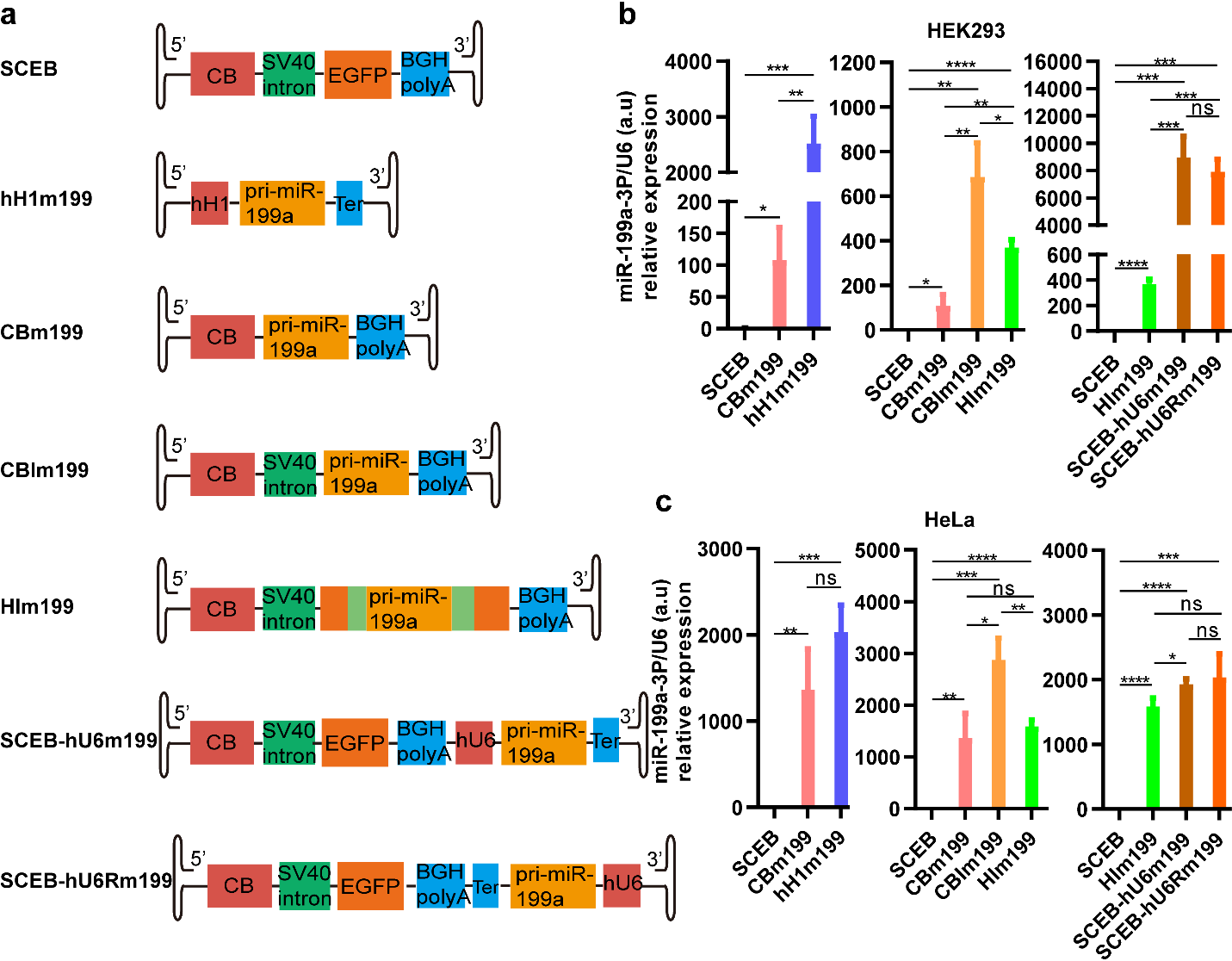
**

**Supplementary Fig. 1** **Comparison of the expression levels of miR-199a in different expression cassettes**

**(a)** Schematic diagram of miR-199a expression cassettes. Two boxes in light green on both sides of pri-miR-199a in the HIm199a vector were indicated as separate parts of another intron named hCG intron from the *hCG* gene. CB, chicken β-actin promoter with CMV enhancer; hH1, human H1 promoter; Ter, TTTTTT sequence, the termination signal for miRs; hU6, human U6 promoter. **(b)** The miR-199a expression levels in HEK293 cells. **(c)** The miR-199a expression levels in HeLa cells. n=3, error bars represent the standard deviation of the mean. **P* <0.05, ***P* <0.01, ****P* <0.001, *****P* <0.0001 and ns represented not significant compared between two groups using the method of two-tailed Student’s t-test.

**
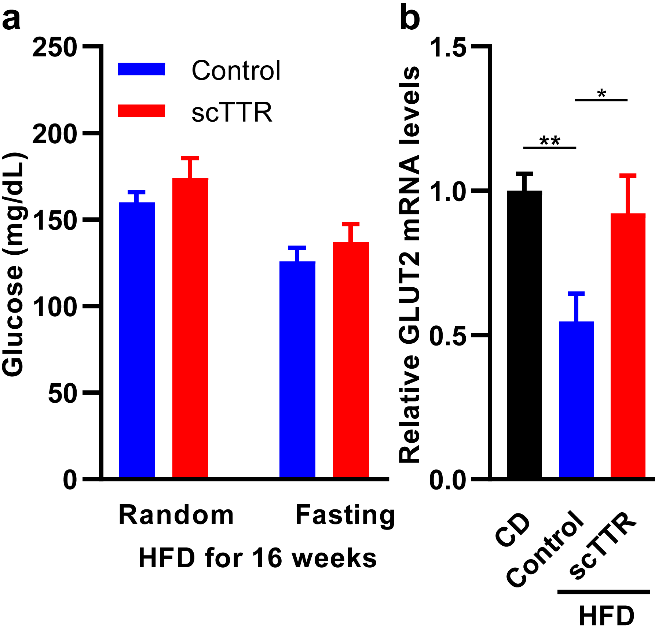
**

**Supplementary Fig. 2 Blood glucose and GLUT2 mRNA expression levels in the liver of mice**

**(a)** Random and fasting blood glucose levels in mice fed a HFD for 16 weeks (n=10 per group). GLUT2, Glucose transporter 2; CD, conventional diet; scTTR, self-complementary AAV-TTR-miR-22 vector; HFD, high-fat diet. **(b)** GLUT2 mRNA levels normalized to 36B4 mRNA levels in the liver of mice after sacrifice (n=6 per group). All data were reported as mean ± SEM. **P* <0.05, ***P* <0.01 compared between two groups using the method of two-tailed Student’s t-test.

**Table 1** The primers for the construction of miR-22 expression cassettes

| **Primer name** | **Primer sequence （5’-3’）** |  |
| --- | --- | --- |
| miR-22-NheⅠ-F | ATCGGATCCGCTAGCTGGCGCCCCCGAGTGGGTGTG |  |
| miR-22-AflⅡ-R | ACCGGATCCTTAAGTTGGTACTGCCACACACAAG |  |
| EGPF-intron-F1-F | GATCCACCGGTCGCCACC |  |
| polyA-KpnⅠ-R | ATCGGTACCCGTACGtcagatctctccccagca |  |
| MCS1-Ter-BsiWⅠ-F | GTACGACTAGTTGCGAGGTACCGCTAGCAATTGCTTAAGTTTTTTC |  |
| MCS1-Ter-BsiWⅠ-R | GTACGAAAAAACTTAAGCAATTGCTAGCGGTACCTCGCAACTAGTC |  |
| hU6-SpeⅠ-F | AccACTAGTaggagggcctatttcccatg |  |
| hU6-KpnⅠ-R | accggtaccagacccggtgtttcgtcc |  |
| mU6-SpeⅠ-F | ACCACTAGTGATCCGACGCCGCCATCTC |  |
| mU6-KpnⅠ-F | ACCGGTACCGCACAAACAAGGCTTTTCTC |  |
| hH1-SpeⅠ-F | ACCACTAGTGAATTCGAACGCTGACGTC |  |
| hH1-KpnⅠ-R | ACCGGTACCTGGGAAAGAGTGGTCTCATAC |  |
| ms-NheⅠ-F | atcGCTAGCcgccagctgatcggagactg |  |
| ms-AflⅡ-R1 | atcCTTAAGctgtcttgtgcctgcctctg |  |
| ms-AflⅡ-R2 | atcCTTAAGcgtgtctttcactgaggctg |  |
| ms-AflⅡ-R3 | atcCTTAAGcaaccccagtcatagatatg |  |
| ss-MCS2-AgeⅠ-F | CCGGTACCGTACGACTAGTGCTAGCAAGCTTAAGAGCT |  |
| ss-MCS2-SacⅠ-R | cttaagcttgctagcactagtcgtacggta |  |
| EGFP-intron-F1-R | caggggccctgcagtcttaccttgatgccgttcttctgc |  |
| EGFP-intron-F2-F | gtaagactgcagggcccctgggcaccttcc |  |
| EGFP-intron-F2-R | ctgggacaaggacactgcttc |  |
| EGFP-intron-F3-F | aagcagtgtccttgtcccaggtgaacttcaagatccgcc |  |
| EGFP-intron-F3-R | tgatcagcgagctctagtcg |  |
| EGint-MCS3-F | agatcttaagcgtacgctagctctagactgca |  |
| EGint-MCS3-R | gtctagagctagcgtacgcttaagatctgca |  |
| miR-199a-NheⅠ-F | accGCTAGccctagtctgctgcaaatgtg | |
| miR-199a-AflⅡ-R | accCTTAAgagggcttcttggtccctag |  |
| m36B4-F | AGATGCAGCAGATCCGCAT |  |
| m36B4-R | GTTCTTGCCCATCAGCACC |  |
| mGLUT2-F | TTCCAGTTCGGCTATGACATCG |  |
| mGLUT2-R | CTGGTGTGACTGTAAGTGGGG |  |

## Supplemental Materials and Methods

### Construction of rAAV vector plasmids

The SCEB plasmid was constructed using the pssAAV-CB-EGFP and the pscAAV-CB-EGFP plasmids, where the EGFP fragment digested by NcoⅠ/SacⅠ double enzymes (New England Biolabs, Ipswich) from pscAAV-CB-EGFP plasmid was cloned into the pssAAV-CB-EGFP vector backbone.

A PCR product of the EGFP-BGH polyA fragment was amplified and then digested by the NcoⅠ/KpnⅠ double enzymes. The digested fragment was cloned into the SCEB vector backbone digested by NcoⅠ/SphⅠ double enzymes. The BsiWⅠ enzyme site to downstream of BGH polyA was placed in the resulting plasmid, which was used for insertion of the multiple cloning sites (MCS1) and termination signal (Ter) of miRNA in the forward or reverse directions to obtain SCEB-MCS1-Ter and SCEB-Ter-MCS1 plasmids. The primers were listed in Supplementary Table 1.

Human U6 promoter (hU6 promoter), mouse U6 promoter (mU6 promoter) and human H1 promoter (hH1 promoter) from HeLa cells or C2C12 cells genome were amplified and cloned into SCEB-MCS1-Ter vector by the SpeⅠ/KpnⅠ double enzymes digestion. Then, the miR-22 sequence (m, 1137 bp) was cloned into the above recombinant plasmid by the NheⅠ/AflⅡ double enzymes digestion. The plasmids for overexpressing EGFP and miR-22 simultaneously including SCEB-hU6, SCEB-mU6 and SCEB-hH1. SCEB-hU6R plasmid was constructed by inserting hU6 promoter and miR-22 sequence into SCEB-Ter-MCS1 plasmid. The primers were listed in Supplementary Table 1.

Three plasmids, including hH1, hU6 and mU6 were constructed using the SCEB vector as the backbone which was digested by the BglⅡ enzyme, and the fragments of hH1-miR-22-Ter, hU6-miR-22-Ter and mU6-miR-22-Ter were obtained from the SCEB-hH1, SCEB-hU6 and SCEB-mU6 plasmids by BsiWⅠ digestion.

ms1-6 rAAV vector plasmids were constructed by using the hH1 vector plasmid as the vector backbone digested by NheⅠ/AflⅡ double enzymes. The six PCR products of miR-22 sequences in different lengths were inserted into the vector backbone. The primers for amplification of miR-22 sequences in different lengths were listed in Supplementary Table 1.

The multiple cloning sites (MCS2) were inserted into the pssAAV-CB-EGFP vector backbone digested by AgeⅠ/SacⅠ double enzymes to construct the pssAAV-CB-MCS2 vector plasmid.

The CB plasmid was constructed using the pssAAV-CB-MCS2 plasmid as the vector backbone. The plasmid was digested by SalⅠ/AflⅡ double enzymes, and the miR-22 sequence digested by NheⅠ/AflⅡ double enzymes was inserted to obtain final recombinant plasmid.

The CBI plasmid was constructed by inserting the miR-22 sequence to pssAAV-CB-MCS2 plasmid vector digested by NheⅠ/AflⅡ double enzymes.

The SI plasmid was constructed by inserting the miR-22 sequence to SCEB plasmid at the BamHⅠ enzyme digestion site in the SV40 intron.

A final PCR product of EGFP1-hCG intron-EGFP2 fragment was obtained following overlap PCR, digestion by NcoⅠ/SacⅠ double enzymes, and subsequently the fragment was cloned into a pscAAV-CB-EGFP vector to construct the pscAAV-CB-EGFP-hCG intron plasmid. Then, the multiple cloning sites (MCS3) were inserted into the hCG intron at PstⅠ site to construct pscAAV-CB-EGFP-hCG-MCS. Next, EGFP-hCG intron-MCS fragment was cloned into the pssAAV-CB-EGFP vector by NcoⅠ/SacⅠ double enzyme digestion. Finally, the miR-22 sequence was inserted into the MCS3 by the NheⅠ/AflⅡ double enzymes digestion to obtain the HI plasmid. The used primers were listed in Supplementary Table 1.

The EG plasmid was constructed using the CBI plasmid as the vector backbone and digested by SalⅠ/HindⅢ double enzymes. The fragment of the SV40 intron-EGFP was digested by SalⅠ/KpnⅠ double enzymes from SCEB plasmid and was cloned into CBI plasmid to obtain the EG plasmid.

The pscAAV-TTR-miR-22-BGH polyA plasmid used for *in vivo* study was constructed by inserting the fragment of the miR-22 sequence digested by SalⅠ/SacⅠ double enzymes from the CB plasmid into the pscAAV-TTR-EGFP-BGH polyA plasmid digested by HpaⅠ/SacⅠ double enzymes.

In this study, the miR-199a sequence contains *Mus musculus* dynamin 3, long non-coding RNA sequence region (NR_002870, 30~428) and was amplified by reverse transcription polymerase chain reaction (RT-PCR) using the mouse liver RNA as the template. All plasmids for miR-199a expression were constructed by replacing miR-22 sequence in the constructed expression vectors with the miR-199a sequence using the NheⅠ/AflⅡ double enzyme digestion. The primers used were listed in Supplementary Table 1.

### RT-qPCR

TRIzol (Invitrogen, Carlsbad) was utilized for total RNA isolation. cDNAs were obtained using 1 μg high-quality RNAs as templates in a 10 μL system to the manufacturer’s protocol (Invitrogen, Carlsbad). The reactions were then placed on a PCR instrument at 37℃ for 50 min, followed by incubation at 70℃ for 15 min to obtain cDNAs.

To measure miR-199a-3P expression levels, each cDNA sample was diluted 20-fold in nuclease-free water for a qPCR reaction to detect miR-199a and U6 snRNA separately in duplicate. A reaction in a 10 μL total volume containing 5 μL of SYBR mix (TransGen Biotech, Beijing), 4.6 μL of diluted cDNA sample, and 0.4 μL of 1 μM miR-199a-3P primer set (GenePharma, Shanghai) or U6 snRNA primer set (GenePharma, Shanghai) was added into PCR tubes (Bio-Rad, Hercules) and the qPCR reaction was performed on an Applied Biosystems instrument. The qPCR procedure was 95℃ for 2 min, followed by 40 cycles of 95℃ for 5 s and 60℃ for 10 s.

To measure GLUT2 mRNA relative levels, each cDNA sample was diluted 100-fold in nuclease-free water for a qPCR reaction to detect GLUT2 and 36B4 mRNA levels separately in duplicate. A reaction in a 10 μL total volume containing 5 μL of SYBR mix (QIAGEN, Hilden), 2.4 μL of diluted cDNA sample, and 0.3 μL of 10 μM GLUT2 primers (TSINGKE, Chengdu) or 36B4 primers (TSINGKE, Chengdu) was added into PCR tubes. The qPCR procedure was 95℃ for 3 min, followed by 40 cycles of 95℃ for 5 s and 60℃ for 15 s.

### Animals

Approximately 6-8 weeks old C57BL/6 male mice (Vital River Laboratory Animal Technology, Beijing) were maintained on a 12-h light-dark cycle.

Mice were treated with different rAAV vectors via tail vein injections. The injection dosage of different rAAVs was 4×10^11^ genome copies (GC) for each mouse. Mice treated with the scTTR rAAV vector and the negative control vector were fed a high-fat diet (Research Diets, New Brunswick) two weeks after rAAV vectors injection, and another group of mice treated with the negative control vector were fed a conventional diet. Livers of mice with rAAV vectors fed a high-fat diet or conventional diet for 35 weeks were collected for measuring GLUT2 mRNA relative levels. In addition, random and fasting blood glucose levels in mice were measured after high-fat diet for 16 weeks.
